# The Role of Mitochondrial Energy Metabolism in the Mechanism of Exercise Improving Depression

**DOI:** 10.3390/cimb47050382

**Published:** 2025-05-21

**Authors:** Yuwei Liu, Chenghao Zhong, Yuxin Yang, Jianbo Hu, Xiaoyan Yi, Jiating Huang, Haonan Li, Xiaojie Liu, Ke Xue, Xianghe Chen

**Affiliations:** College of Physical Education, Yangzhou University, Yangzhou 225009, China; yangdayw@163.com (Y.L.); 18950056613@163.com (C.Z.); yyx781535082@163.com (Y.Y.); 18822098452@163.com (J.H.); 15576961328@163.com (X.Y.); huangjiating8012@163.com (J.H.); lisi_9527@163.com (H.L.); liuxiaojie88888888@163.com (X.L.); 13775633406@163.com (K.X.)

**Keywords:** mitochondria, energy metabolism, mitochondrial function, exercise, depression

## Abstract

Depression is the most disabling neuropsychiatric disorder, but its exact mechanisms remain unclear. Mitochondrial energy metabolism may play a key role in the onset and development of depression. Cytokines such as PGC-1α, NLRP3, and BDNF can influence mitochondrial energy metabolism by regulating mitochondrial biogenesis, immune inflammation, and neuroplasticity, thereby mediating the occurrence and progression of depression. Exercise can improve depression by regulating mitochondrial energy metabolism. The molecular mechanisms are closely related to the upregulation of exercise-induced PGC-1α, AMPK, SIRT1, and BDNF expression, as well as the downregulation of NLRP3 expression. These factors can activate key factors or pathways such as Nrf2, AMPK, and PKA/CREB, while inhibiting the excessive activation of NF-κB. Through these mechanisms, they regulate the expression of downstream target genes (such as TFAM, NRF1, CREB, and Bcl-2), thereby enhancing mitochondrial biogenesis and improving the quantity and quality of mitochondria. Additionally, they can act to inhibit the release of inflammatory factors to improve immune inflammation, enhance neuroplasticity, promote neuronal growth, and facilitate synapse formation and remodeling, thereby enhancing mitochondrial energy metabolism and improving its dysfunction, which in turn alleviates depression. Currently, there is a lack of systematic and comprehensive research on the mechanisms by which exercise improves depression through mitochondrial energy metabolism. Therefore, this article aims to review and analyze the role of mitochondrial energy metabolism in the improvement of depression through exercise, in order to provide a new theoretical basis and research ideas for the prevention and treatment of depression.

## 1. Introduction

Depression is a severe mental disorder characterized by persistent low mood, loss of pleasure, and cognitive dysfunction, and it is one of the leading causes of disability worldwide [1]. The occurrence of depression is influenced by both genetic predisposition and environmental factors. The known mechanisms of onset mainly include the monoamine neurotransmitter hypothesis, the hypothalamus–pituitary–adrenal (HPA) axis hypothesis, immune activation and suppression, and the norepinephrine theory, among others [2]. In recent years, an increasing number of studies have focused on the relationship between energy metabolism and depression. The onset of depression is closely related to abnormalities in energy metabolism, which primarily occur in the mitochondria. Research has confirmed that mitochondrial energy metabolism abnormalities are an important mechanism leading to the occurrence of depression [3].

Research has assessed the metabolic rates of the prefrontal cortex and basal ganglia in patients with depression, finding that their brain energy metabolism and glucose metabolism levels are low [4,5]. Patients with Major Depressive Disorder (MDD) often exhibit mitochondrial energy metabolism disorders and brain energy imbalances, with lower levels of adenosine triphosphate (ATP) in the brain [6,7]. Additionally, a reduction in the number of mitochondria and ATP production in the muscle tissues of animal models of depression is also believed to be related to symptoms such as fatigue and low motivation [8], which are common in animal models of depression [9]. Currently, energy metabolism disorders caused by mitochondrial dysfunction have become one of the main reasons for the pathogenesis of depression and other neuropsychiatric diseases [10]. Mitochondrial dysfunction is closely linked to key signaling pathways such as peroxisome proliferator-activated receptor gamma coactivator-1 alpha (PGC-1α), Sirtuin 1 (SIRT1), NOD-like receptor family pyrin domain containing 3 (NLRP3), and Brain-derived neurotrophic factor (BDNF) [11,12]. SIRT1 enhances the activity of PGC-1α through deacetylation, and together they regulate mitochondrial biogenesis; insufficient expression of either can affect mitochondrial quantity and function, leading to neuronal energy supply disorders [13]. Insufficient energy supply can inhibit the expression of BDNF, which is crucial for neuronal survival, development, and synaptic plasticity. Furthermore, mitochondrial dysfunction may also lead to the activation of the NLRP3 inflammasome, further promoting the release of cytokines such as Interleukin-1β (IL-1β) and IL-18, exacerbating damage to neuronal function [14,15]. Notably, SIRT1, as an NAD+-dependent deacetylase, is involved in regulating cellular metabolism, stress responses, and inflammatory responses, playing an important role in maintaining mitochondrial function and promoting cell survival. Impaired expression of both SIRT1 and PGC-1α may exacerbate the decline in mitochondrial function, leading to metabolic disorders, which could potentially trigger depression [16,17,18].

Exercise is an important means to improve depression [19,20]. It has been reported that exercise may alleviate depressive behaviors by enhancing the expression levels of PGC-1α in skeletal muscle and hippocampal tissue [21,22]. In a study involving 61 depressed college students, a 6-week exercise intervention resulted in a reduction of depressive symptoms and levels of inflammatory factors such as tumor necrosis factor-α (TNF-α), IL-6, and IL-1β [23]. The activation of the NLRP3 inflammasome was found to enhance the expression of these inflammatory factors [24]. This suggests that moderate to high-intensity exercise may improve depressive moods through anti-inflammatory effects. Additionally, after 6 weeks of running wheel exercise, the levels of neurotrophic factors such as BDNF increased, and hippocampal volume also increased, leading to a reduction in depressive-like behaviors [25]. However, blocking the expression of BDNF in the hippocampus can weaken the neuroplastic effects regulated by exercise, thereby inducing the occurrence and development of depression [26].

Research indicates that exercise can alleviate depressive symptoms by regulating mitochondrial function [27]. Mitochondrial energy metabolism plays a crucial role in the occurrence and development of depression, and exercise, as a non-pharmacological intervention, can effectively improve depression. The specific mechanisms may include exercise upregulating the expression of PGC-1α and BDNF to promote mitochondrial biogenesis and neuroplasticity. Meanwhile, exercise inhibits the activation of the NLRP3 inflammasome to reduce inflammatory responses, thereby improving mitochondrial energy metabolism and ultimately alleviating depression. Currently, most existing research focuses on regulating mitochondrial dysfunction to screen for effective therapies. Although the improvement of depression through exercise has been confirmed, the emphasis has mainly been on the effects of exercise on neurotransmitters (such as serotonin and dopamine) and the neuroendocrine system, while the core mechanisms of mitochondrial energy metabolism in this process have not been systematically reviewed. Based on this, the aim of this study is to comprehensively outline the interaction mechanisms between depression and mitochondria, and to summarize the key molecular pathways regarding how exercise regulates mitochondrial energy metabolism to improve depression, in order to provide theoretical support and research direction for understanding the role of mitochondrial energy metabolism in the mechanism by which exercise alleviates depression.

## 2. Method

This study conducts a narrative review and implements a non-systematic literature review, focusing on the mechanisms by which mitochondrial energy metabolism improves depression through exercise. To narrow down the research topic, we selected the most relevant original studies, clinical trials, meta-analyses, and reviews from Chinese and English literature up to October 2024. The search keywords included (used alone or in combination): mitochondria, energy metabolism, mitochondrial function, exercise, depression, biogenesis, neuroinflammation, neuroplasticity, and neurogenesis. The literature search databases included PubMed, CNKI (China National Knowledge Infrastructure), and Web of Science.

**Figure 1:**
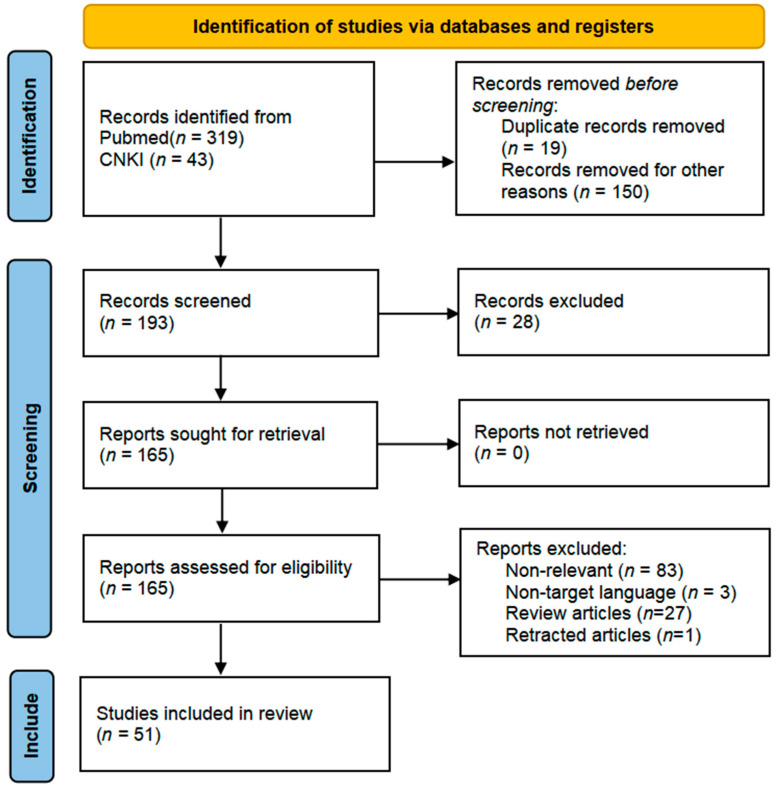
The PRISMA 2020 flow diagram was used for the identification of the studies included in this review. No automation tools were used for the screening process.

The research followed the PRISMA guidelines for systematic literature screening, with the specific process as follows: a total of 362 potentially relevant articles were obtained through systematic searches. A three-tier screening mechanism was implemented based on predefined inclusion criteria: first, a preliminary screening of titles/abstracts was conducted, with inclusion criteria including (1) peer-reviewed original research or systematic reviews; (2) a clear discussion of the mechanisms by which mitochondrial energy metabolism improves depression; (3) inclusion of well-designed in vitro experiments, animal models, or clinical trials. A total of 169 articles were excluded in the preliminary screening, including duplicate publications (*n* = 19), non-research literature such as conference abstracts (*n* = 38), and case reports or other literature with insufficient evidence levels (*n* = 112). Subsequently, 193 articles underwent methodological quality assessment, leading to the exclusion of 28 articles with significant flaws in experimental design or insufficient relevance to the mechanisms of mitochondrial energy metabolism improving depression. The remaining 165 articles underwent full-text review, focusing on excluding the following three categories: (1) studies focusing on other antidepressant mechanisms (*n* = 51); (2) studies with comorbid interference in the subject population (*n* = 35); (3) review articles and retracted literature (*n* = 28). Ultimately, 51 articles met all inclusion criteria (Figure 1).

## 3. The Role of Mitochondrial Energy Metabolism in the Mechanism of Depression

### 3.1. Mitochondrial Energy Metabolism Improves Depression by Regulating Biogenesis

Mitochondrial biogenesis refers to the process of generating new mitochondria to maintain their quantity and function [28,29]. As a core regulatory factor, PGC-1α participates in the pathological process of depression by promoting energy metabolism and neurogenesis [14,30,31]. When the expression of PGC-1α and its downstream target genes TFAM, NRF1/2 is suppressed, it affects the replication and transcription of mtDNA, leading to impaired mitochondrial biogenesis, which induces mitochondrial dysfunction and further triggers energy metabolism disorders and oxidative stress, mediating the onset of depression [32]. Clinical evidence has found that the expression of PGC-1α and downstream genes TFAM and NRF1 is downregulated in the blood monocytes of patients with Major Depressive Disorder (MDD) [33]. Animal experiments further indicate that upstream target genes of PGC-1α, such as AMP-activated protein kinase (AMPK) and SIRT1, are significantly decreased, thereby mediating the occurrence and development of depression [34]. Additionally, a study in an animal model of depression induced by prenatal stress found that the reduced level of PGC-1α protein in the hippocampus inhibits mitochondrial biogenesis through the PGC-1α/NRF1/TFAM pathway, leading to depressive-like behavior [35]. Mitochondrial biogenesis is primarily regulated by the SIRT1/PGC-1α and AMPK/PGC-1α signaling pathways, with AMPK and SIRT1 being key factors in regulating mitochondrial biogenesis [36]. As an upstream regulatory factor, AMPK directly phosphorylates threonine-177/serine-538 residues on PGC-1α, while also indirectly regulating its deacetylation to activate PGC-1α by enhancing SIRT1 activity (dependent on NAD+ levels) [37,38]. Activated PGC-1α cooperates with NRF1/2 to upregulate TFAM expression [39], promoting the replication of mitochondrial DNA (mtDNA) and protein synthesis through the PGC-1α-NRF1/2-TFAM pathway, ultimately facilitating mitochondrial biogenesis and alleviating depression [14,40].

Another study found that insulin-like growth factor 1 (IGF-1) can upregulate the expression of PGC-1α by activating the PI3K/Akt signaling pathway, thereby promoting mitochondrial biogenesis and alleviating depressive-like behavior [41,42,43,44]. Notably, a deficiency in IGF-1 can lead to mitochondrial fragmentation, often accompanied by dysfunction, reduced ATP production, and oxidative stress, ultimately mediating the onset of depression through impaired energy metabolism [45]. Research by Yang et al. found that a high-fat diet induces mitochondrial dysfunction in the hippocampus by inhibiting the CREB/PGC-1α signaling pathway, which in turn triggers depressive-like behavior [46]. IGF-1 can activate the CREB/PGC-1α signaling pathway to regulate the expression of genes such as NRF1, TFAM, and the mitochondrial dynamics-related protein Drp1, promoting mitochondrial biogenesis and improving function, ultimately alleviating depression [47,48,49,50]. This suggests that the CREB/PGC-1α signaling pathway may be an important molecular mechanism through which IGF-1 improves depression by regulating mitochondrial function. Fibroblast growth factor 21 (FGF21), a key factor in regulating energy metabolism and protecting the nervous system, exerts antidepressant effects by multi-target regulation of mitochondrial function [51]. After crossing the blood-brain barrier, it binds to the FGF receptor complex and enhances PGC-1α activity by activating the AMPK/SIRT1 pathway, thereby promoting the expression of downstream transcription factors NRF1/2 and facilitating mitochondrial biogenesis in the midbrain [52]. At the same time, this molecule upregulates the expression of heme oxygenase-1 (HO-1) through the NRF2 signaling axis, thereby enhancing antioxidant enzyme activity [53]. This process helps maintain the physiological levels of reactive oxygen species (ROS) and improves mitochondrial function. Ultimately, by promoting the synergistic effects of mitochondrial biogenesis and redox homeostasis, this molecule improves depressive-like behavior [54]. Currently, numerous studies have demonstrated that mitochondrial biogenesis involves a complex network of signaling pathways, including key regulatory factors such as PGC-1α, NRF1, and NRF2. However, the differences in their roles across different tissues, especially in various regions of the brain, remain to be revealed. AMPK, as a key regulatory factor in energy metabolism, has been shown to promote mitochondrial biogenesis by activating PGC-1α. However, the interaction mechanisms of AMPK with other signaling pathways, such as the mammalian target of rapamycin (mTOR) and PI3K/Akt, and their roles in regulating mitochondrial energy metabolism and depression have not been fully elucidated. This area warrants further investigation in the future.

### 3.2. Mitochondrial Energy Metabolism Improves Depression by Mediating Immune Inflammation

Patients with depression exhibit elevated levels of pro-inflammatory cytokines (IL-6, IL-8), interferon-gamma (IFN-γ), and TNF-α [55]. The NLRP3 inflammasome, a key pathway for the production of pro-inflammatory factors, shows significant activation in the peripheral blood mononuclear cells and brains of both depressed patients and LPS-induced depressed mice, accompanied by increased levels of IL-1β. This suggests that it is a core target mediating immune activation and the onset of depression [56,57]. Abnormal activation of the NLRP3 inflammasome is associated with the occurrence of depression [58]. Studies have found that activation of the NLRP3/caspase-1/Gasdermin D (GSDMD) pathway leads to pyroptosis of astrocytes in the hippocampus of chronically stressed mice, enhancing neuroinflammatory responses and mediating the onset of depression [59]. Analyzing the mechanism, under chronic stress conditions, cells experience oxidative stress and mitochondrial dysfunction. The released mtDNA, mitochondrial reactive oxygen species (mtROS), and cardiolipin promote the expression of the NLRP3 inflammasome, activating caspase-1 and cleaving GSDMD, which triggers pyroptosis [60]. In this process, pro-inflammatory cytokines IL-1β and IL-18 are also activated by caspase-1, enhancing the inflammatory response [61]. Additionally, various damage-associated molecular patterns (DAMPs, such as NF-κB, IL-1β, ROS, and oxidized mtDNA) activate the NLRP3 inflammasome, upregulating the activity of indoleamine 2,3-dioxygenase (IDO), which shifts tryptophan metabolism towards the kynurenine pathway, thereby inhibiting serotonin synthesis [62,63]. In the central nervous system, kynurenine can be further converted into quinolinic acid (QA), and the abnormally elevated levels of QA in the brains of depressed patients promote excessive release of glutamate and inhibit its reuptake, leading to neurotoxicity and mitochondrial dysfunction, which exacerbates oxidative stress and the accumulation of oxidized mtDNA [64,65]. These changes can reactivate the NLRP3 inflammasome, forming a pro-inflammatory feedback loop that intensifies the expression of inflammatory factors. The increase in inflammatory factors (such as IL-6, IL-8, IL-1β, and TNF-α) activates the immune system, increases oxidative stress and neuroinflammation, resulting in excessive ROS production [66]. Among these, TNF-α impairs mitochondrial metabolism by increasing ROS production and inhibiting the electron transport chain complex IV, leading to reduced mitochondrial membrane potential and ATP generation, damaging mitochondrial respiratory chain function, and causing mitochondrial energy metabolism disorders that trigger depression [67,68,69]. Other studies have found that in chronic stress-induced depression models, knockout of uncoupling protein 2 (UCP2) leads to mitochondrial dysfunction, abnormal mitochondrial membrane potential, and overload of the electron transport chain, resulting in ROS accumulation and oxidative stress [70,71], promoting the activation of the ROS-TXNIP-NLRP3 inflammasome axis, which causes the release of pro-inflammatory factors such as IL-1β, leading to impaired neurogenesis, loss of astrocytes, and mitochondrial energy metabolism disorders, ultimately mediating the onset of depression [72].

The cGAS-STING signaling pathway is involved in the occurrence of various autoimmune, inflammatory, and psychiatric diseases [73]. Mitochondrial dysfunction leads to the release of mtDNA into the cytoplasm, activating this pathway [74]. This process promotes the phosphorylation of downstream key molecules TBK1 and IRF3. Subsequently, IRF3 enters the nucleus, regulating the production and release of inflammatory factors such as type I interferon β (IFN-β), TNF-α, and IL-6, enhancing the inflammatory response, which in turn affects neuronal survival and neuronal regeneration, especially in hippocampal neurogenesis [75,76,77]. Furthermore, the sustained activation of the cGAS-STING pathway can also increase the production of ROS, leading to more mtDNA release, which further drives the secretion of IL-1β and IL-18 and affects ATP synthesis [78,79], impacting mitochondrial energy metabolism and mediating the onset of depression [80]. This study found that immune inflammation mediates the occurrence of depression by affecting mitochondrial energy metabolism. However, it is important to note that neuroinflammation and mitochondrial dysfunction may form a vicious cycle through the release of damage-associated molecular patterns (DAMPs) [14]. Therefore, the relationship between inflammation-mediated depression and mitochondrial dysfunction warrants further investigation.

### 3.3. Mitochondrial Energy Metabolism Improves Depression by Promoting Neural Plasticity

**Figure 2:**
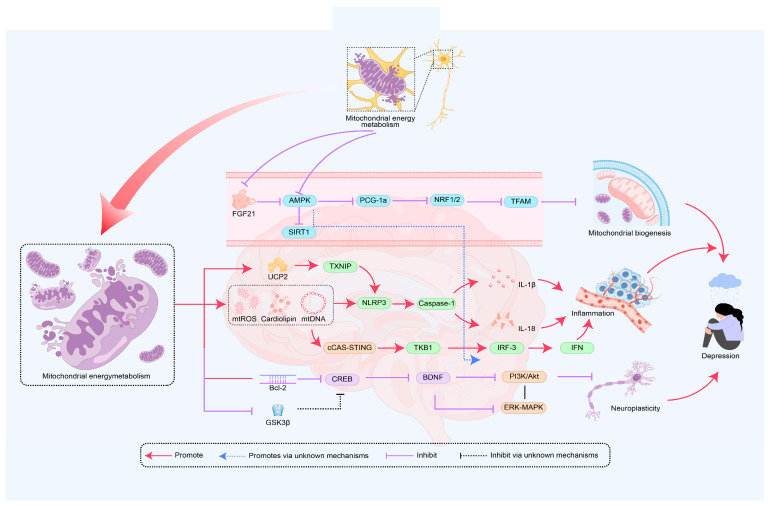
The role of mitochondrial energy metabolism in the mechanism of depression onset. The disruption of mitochondrial energy metabolism is regulated by mitochondrial biogenesis, inflammation, and neural plasticity, with key signaling pathways including PGC-1α, AMPK, NLRP3, and BDNF. AMPK—AMP-activated protein kinase; PGC-1α—Peroxisome proliferator-activated receptor gamma coactivator 1-alpha; NRF1—Nuclear respiratory factor 1; NRF2—Nuclear factor erythroid 2-related factor 2; TFAM—Transcription factor A, mitochondrial; SIRT1—Sirtuin 1; NLRP3—NOD-like receptor family pyrin domain containing 3; Caspase-1—Cysteine-dependent aspartate-specific protease 1; IL-6—Interleukin-6; IL-18—Interleukin 18; UCP2—Uncoupling protein 2; TXNIP—Thioredoxin-interacting protein; IRF3—Interferon regulatory factor 3; IFN—Interferon; PI3k—Phosphoinositide-3 kinase; cGAS—Cyclic GMP-AMP Synthase; STING—Stimulator of Interferon Genes; Bcl-2—B-cell lymphoma 2; CREB—cAMP response element-binding protein; BDNF—Brain-derived neurotrophic factor; GSK3β—Glycogen synthase kinase 3 beta; PI3K—Phosphatidylinositol 3-Kinase; ERK—Extracellular signal-regulated kinase; Multiple pathways mediate the occurrence of depression through mitochondrial energy metabolism.

Most patients with MDD exhibit mitochondrial energy metabolism disorders, which may be closely related to reduced neural plasticity and impaired hippocampal neurogenesis [7]. The chronic unpredictable mild stress (CUMS) combined with sleep deprivation depression model shows that dendritic spine damage in the dentate gyrus (DG) of the hippocampus is accompanied by neural network disruption, significantly inhibiting neural plasticity [81]. As a core regulatory factor of neural plasticity, BDNF promotes synaptic plasticity by enhancing mitochondrial energy metabolism and neuronal glucose utilization [82]. It is worth noting that mitochondrial dysfunction can negatively affect the expression of BDNF. The downregulation of BDNF expression can impair synaptic remodeling and neuronal connectivity, affect neural plasticity, exacerbate issues related to energy metabolism disorders, and subsequently mediate the development of depression [83,84]. Mechanistically, BDNF exerts neuroprotective effects through the Mitogen-activated protein kinase kinase/B-cell lymphoma 2 (MEK/Bcl2) signaling pathway [85], while cAMP response element-binding protein (CREB), as a downstream transcription factor of the BDNF pathway, is associated with reduced activity in the occurrence of depression [86]. Mitochondrial dysfunction can lead to oxidative stress that inhibits the Bcl-2-related signaling pathway, resulting in decreased CREB transcriptional activity and downregulation of BDNF expression, which in turn reduces the activity of the PI3K/Akt and extracellular regulated protein kinase/mitogen-activated protein kinase (ERK/MAPK) pathways, thereby weakening cellular antioxidant stress and energy metabolism [11], affecting neural plasticity and mediating the onset of depression [87]. Additionally, neural progenitor mice lacking Tropomyosin receptor kinase B (TrkB) exhibit decreased proliferation of neural progenitor cells and impaired neuronal survival, leading to impaired neurogenesis and depressive-like behavior, with TrkB knockout mice becoming insensitive to antidepressant treatment in depressive-like behavior [88]. This research highlights the important role of TrkB in emotional regulation and underscores the significance of neural plasticity regulatory mechanisms in the pathological process of depression. Research has found that the main active peptide component of bee venom, Mel, can induce depressive-like behavior in mice. Mel leads to impaired hippocampal synaptic plasticity and mitochondrial dysfunction through the BDNF/TrkB/CREB signaling pathway [89]. Specifically, Mel inhibits the release of BDNF, limits the binding and activation of TrkB receptors, and subsequently suppresses the phosphorylation of CREB, resulting in damage to the downstream PI3K/Akt and MAPK/ERK signaling pathways [90]. This affects the functional shaping of neural networks, leads to impaired neural plasticity, and impacts mitochondrial energy metabolism, which may mediate the occurrence of depressive-like behavior [91]. Research has found that photobiomodulation (PBM) is a method that influences brain activity, functional connectivity, and plasticity through a certain duration of sunlight or artificial light exposure. Studies on CUMS mice and a cortisone-induced hippocampal neuron damage model have shown that PBM can upregulate the expression of BDNF. This process helps restore synaptic function in the hippocampus, reduces oxidative stress, and improves mitochondrial function, alleviating depressive-like behavior [92]. These studies indicate that mitochondrial dysfunction plays an important role in the impaired neurogenesis and neural plasticity associated with the onset of depression. Mitochondrial dysfunction affects energy metabolism through its impact on BDNF and related signaling pathways, mediating the occurrence and development of depression. Although the hippocampus and prefrontal cortex are considered crucial in depression, our understanding of how mitochondrial function in these brain regions specifically affects changes in neural plasticity is still limited. Additionally, GSK-3β is currently believed to have a dual role in regulating cellular energy metabolism and neural plasticity. Changes in its activity may affect the BDNF and CREB signaling pathways, mediating the onset of depression, but the specific mechanisms and pathways remain to be further studied (Figure 2).

## 4. The Role of Mitochondrial Energy Metabolism in Exercise-Induced Improvement of Depression

### 4.1. Mitochondrial Energy Metabolism Mediates the Improvement of Depression Through Biogenesis Induced by Exercise

Energy metabolism disorder is a key factor in the occurrence of depression. The reduction of PGC-1α levels can exacerbate mitochondrial dysfunction by affecting mitochondrial biogenesis, leading to energy metabolism disorders and mediating the occurrence of depression [93]. Exercise can significantly improve depression, and its mechanism is closely related to the enhancement of mitochondrial biogenesis through the AMPK, PGC-1α, and SIRT1 signaling pathways [2]. However, the effects of different types of exercise on outcomes and the systematic evaluation of long-term exercise interventions remain to be revealed. AMPK is a core factor in maintaining energy homeostasis, and its main function is closely related to ATP levels [94]. Exercise increases ATP consumption, raising the AMP/ATP ratio, which activates AMPK and promotes the upregulation of PGC-1α expression [95]. Through the AMPK/PGC-1α signaling pathway, the expression of mitochondrial-related genes such as NRF1 and TFAM is upregulated, promoting mtDNA replication and the formation of new mitochondria, thereby enhancing mitochondrial biogenesis in response to increased energy demand and alleviating depressive symptoms [96,97,98]. Research has found that CUMS mice exhibit reduced levels of PGC-1α along with depressive-like behaviors. Aerobic exercise increases the expression of PGC-1α by enhancing p-AMPK activity, which raises the ratio of p-AMPK to AMPK and increases ATP content. This promotes an increase in the content of mitochondrial primary transcripts mRNA and mtDNA. This process can enhance mitochondrial biogenesis, improve energy metabolism status, and alleviate depressive-like behaviors in chronically mildly stressed mice [19,99]. However, when PGC-1α is knocked out, the adaptive capacity for exercise-induced mitochondrial synthesis and energy metabolism is weakened [39]. SIRT1 plays a key role in regulating mitochondrial biogenesis. Its absence in neurons of the prefrontal cortex and hippocampus can lead to impaired mitochondrial function and induce depressive-like behavior [100]. Exercise induces SIRT1 activation by upregulating AMPK expression and increasing NAD+ levels in cells. This activation promotes the deacetylation of PGC-1α and its downstream transcription factors NRF1/2, enhancing mtDNA transcription. Through the SIRT1/PGC-1α signaling pathway, this process increases the number and function of mitochondria, thereby improving mitochondrial energy metabolism and alleviating the occurrence of depression [101,102,103]. SIRT1 activates PGC-1α through deacetylation and cooperatively regulates the transcription factors FOXO1/FOXO3, upregulating the expression of downstream genes such as manganese superoxide dismutase (MnSOD) and catalase (CAT), which are antioxidant enzymes. This also enhances the function of the electron transport chain and ATP synthesis, promoting metabolic regulation and antioxidant defense [104]. This process alleviates depression by reducing the accumulation of ROS and improving mitochondrial function [105]. Other studies have found that exercise can enhance mitochondrial biogenesis by upregulating the expression of IGF-1 and activating the PI3K/Akt signaling pathway, as well as upregulating the expression of PGC-1α [106]. Additionally, exercise reduces the apoptosis of nerve cells through the Akt signaling pathway, increases their proliferation and survival, exerts neuroprotective effects by regulating mitochondrial function, promotes ATP production, improves energy metabolism, and alleviates depression [107,108]. Exercise can also activate the AMPK and peroxisome proliferator-activated receptor alpha (PPARα) pathways, upregulating FGF21 expression and activating the downstream factor PGC-1α to enhance mitochondrial biogenesis and fatty acid oxidation, improving mitochondrial energy metabolism to alleviate depression [109]. In summary, exercise enhances mitochondrial biogenesis and improves energy metabolism through signaling pathways such as AMPK/PGC-1α and SIRT1, thereby exerting antidepressant effects. However, there are still shortcomings in revealing the mechanisms, and future research could explore the dynamic changes in the interactions between AMPK, SIRT1, and PGC-1α under different exercise intensities and durations. Additionally, current research on the aforementioned signaling pathways presents contradictory results, which may be due to specific effects in certain brain regions, cell types, downstream signaling pathways, and differences in the mouse models used. Therefore, further in-depth research is needed to reveal these complexities.

### 4.2. Mitochondrial Energy Metabolism Mediates the Improvement of Depression Through Inflammatory Responses Induced by Exercise

**Figure 3:**
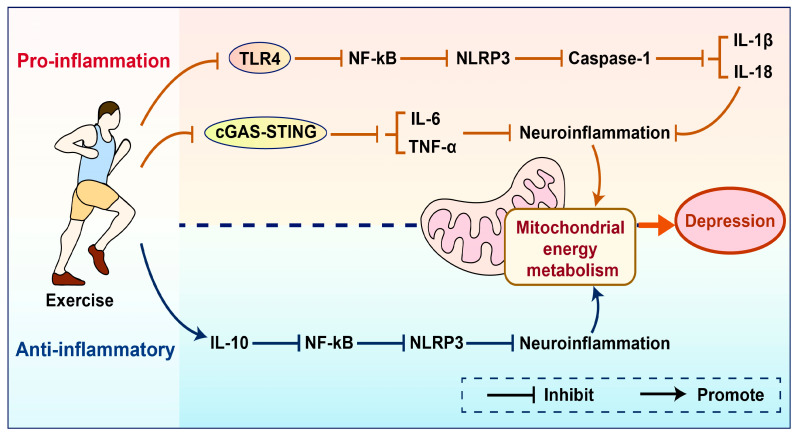
Schematic comparison of pro-inflammatory/anti-inflammatory effects mediated by exercise.

The activation of NLRP3 can lead to the release of pro-inflammatory factors IL-1β and IL-18, triggering an inflammatory response. Exercise can significantly reduce the expression of inflammatory cytokines TNF-α, IL-1β, and IL-6 induced by metabolic disorders [110], thereby inhibiting oxidative stress and microglia-induced neuroinflammation, and improving mitochondrial dysfunction [111]. The activation of NLRP3 is primarily due to mitochondrial dysfunction and excessive production of ROS, with NF-κB being one of the important factors affecting mitochondrial function and a core mediator of the inflammatory process [112]. Therefore, regulating the NF-κB signaling pathway may be one of the key mechanisms by which the NLRP3 inflammasome mediates the prevention and treatment of depression through exercise [113]. This study reveals the relationship between NLRP3 inflammasome activation and mitochondrial dysfunction, clarifying the role of NF-κB as a key regulatory factor, and providing an important theoretical basis for the role of exercise in improving inflammation and mitochondrial energy metabolism. Mechanistically, exercise enhances the activity of antioxidant enzymes such as superoxide dismutase (SOD) and glutathione peroxidase (GPx) by improving mitochondrial function, thereby reducing excessive ROS production and alleviating oxidative stress [114]. Meanwhile, exercise can also upregulate the expression of the anti-inflammatory factor IL-10, regulate the NF-κB and NLRP3 signaling pathways, and inhibit the expression of inflammatory factors TNF-α, IL-6, and IL-1β. These regulatory effects collectively restore neural balance and improve depressive symptoms [115]. Research on humans has found that regular exercise can reduce the generation of reactive oxygen species (ROS) and downregulate the expression of NLRP3 by inhibiting the Toll-like receptor 4 (TLR4)/NF-κB pathway. This, in turn, suppresses the activation of caspase-1 and decreases the production of IL-1β and IL-18, thereby improving mitochondrial energy metabolism and alleviating depression [116]. Other studies have found that exercise can reduce the generation of ROS by upregulating the expression of UCP2 and lowering the mitochondrial membrane potential, thereby inhibiting the activation of the NF-κB and TXNIP/NLRP3 inflammasome pathways, reducing the release of pro-inflammatory factors (such as IL-1β and IL-18), and ultimately improving mitochondrial energy metabolism and alleviating depression-related symptoms [22,117,118]. Mitochondrial dysfunction leads to the release of mtDNA and increased ROS, activating the STING1 and NLRP3 inflammasome signaling pathways, promoting the synthesis of inflammatory factors, and consequently triggering inflammation that mediates the occurrence of depression [119]. Exercise alleviates the inflammatory response by reducing the release of mtDNA and ROS in the cytoplasm. Furthermore, exercise may inhibit the activation of the STING signaling pathway by regulating the expression of cGAS and the synthesis of cGAMP, thereby inhibiting the activity of immune response signaling molecules TBK1 and IRF3 [120]. By inhibiting the cGAS-STING pathway, exercise reduces the expression of interferon beta-1 (IFNβ1) and other pro-inflammatory cytokines IL-6 and TNF-α, thereby decreasing the inflammatory response, improving mitochondrial function, and alleviating depression [121]. In summary, exercise regulates signaling pathways such as NF-κB, NLRP3, UCP2, and cGAS-STING, reduces the release of mtDNA and pro-inflammatory factors, inhibits the inflammatory response, and decreases ROS production, thereby improving mitochondrial function and alleviating depression. However, there are still many blind spots. Existing research has indicated that the activation of the JAK/STAT signaling pathway is related to changes in mitochondrial energy metabolism [122]. It remains to be explored whether exercise may influence neuroinflammation and mitochondrial energy metabolism by regulating JAK/STAT signaling, thereby mediating the occurrence of depression, and the specific mechanisms require further in-depth investigation (Figure 3).

Exercise can inhibit the expression of inflammatory factors by suppressing key signaling pathways such as TLR4 and cGAS-STING. It can also upregulate the expression of the anti-inflammatory factor IL-10, thereby reducing neuroinflammation, improving mitochondrial energy metabolism, and alleviating depression. TLR4—Toll-like receptor; NF-κB—Nuclear Factor kappa-light-chain-enhancer of activated B cells; NLRP3—NOD-like receptor family pyrin domain containing; Caspase-1—Cysteine-dependent aspartate-specific protease 1; IL-18—Interleukin 18; IL-1β—Interleukin-1 beta; cGAS—Cyclic GMP-AMP Synthase; STING—Stimulator of Interferon Genes; IL-6—Interleukin-6; TNF-α—Tumor Necrosis Factor-alpha; IL-10—Interleukin-10; NLRP3—NOD-like receptor family pyrin domain containing 3.

### 4.3. Mitochondrial Energy Metabolism Mediates Exercise-Induced Improvement of Depression Through Neural Plasticity

**Figure 4:**
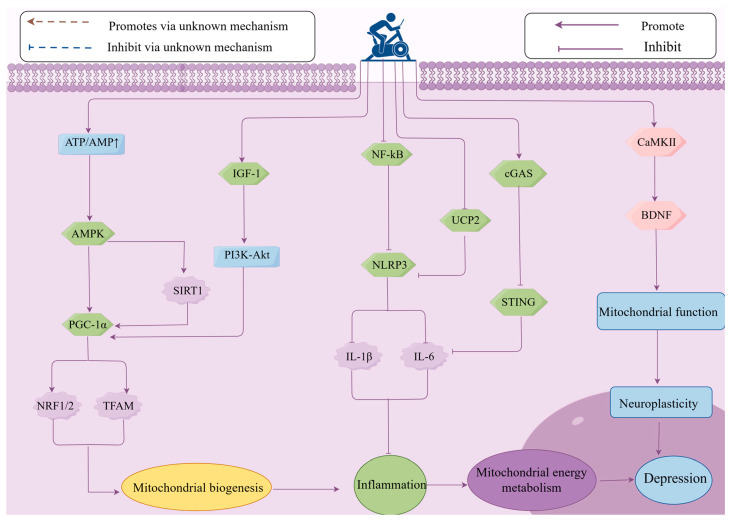
The role of mitochondrial energy metabolism in the mechanism of exercise in improving depression. Exercise can alleviate mitochondrial energy metabolism and improve depression by regulating the expression of key signaling pathways, such as upregulating AMPK, PGC-1α, IGF-1, BDNF, and downregulating NLRP3 and cGAS-SING, thereby influencing mitochondrial biogenesis, inflammation, and neuroplasticity. ATP—Adenosine Triphosphate; AMP—Adenosine Monophosphate; AMPK—AMP-activated protein kinase; PGC-1α—Peroxisome proliferator-activated receptor gamma coactivator 1-alpha; NRF1—Nuclear respiratory factor 1; NRF2—Nuclear factor erythroid 2-related factor; TFAM—Transcription factor A, mitochondrial; SIRT1—Sirtuin 1; IGF-1—Insulin-like Growth Factor 1; PI3K—Phosphoinositide 3-Kinase; Akt—AK strain Transforming; NF-κB—Nuclear Factor kappa-light-chain-enhancer of activated B cells; NLRP3—NOD-like receptor family pyrin domain containing 3; IL-6—Interleukin-6; IL-1β—Interleukin-1 beta; UCP2—Uncoupling protein 2; cGAS—Cyclic GMP-AMP Synthase; STING—Stimulator of Interferon Genes; CaMKII—Calcium/Calmodulin-Dependent Protein Kinase II; BDNF—Brain-derived neurotrophic factor.

Mitochondrial dysfunction can lead to weakened neural plasticity and neural damage, such as energy metabolism disorders, thereby affecting the occurrence and development of depression [85]. There is evidence that impaired neural plasticity in patients with MDD is closely related to mitochondrial dysfunction, while exercise enhances neural plasticity by improving mitochondrial function [123]. BDNF is a key factor in regulating neural plasticity. Exercise can upregulate BDNF expression by stimulating neuronal regeneration and activating the AMPK and Calcium/Calmodulin-dependent protein kinase II (CaMKII) signaling pathways, promoting the production of cytokines and proteins involved in synaptic remodeling, and improving mitochondrial function, thereby enhancing neural plasticity and alleviating depression [124]. CREB is a convergence point for signaling pathways that partially regulate enhanced synaptic activity (such as CaMKII and MAPK) [125]. Exercise triggers calcium influx by activating the CaMKII signaling pathway, promoting CREB phosphorylation, and inducing BDNF expression [44,126]. BDNF activates downstream signaling pathways such as MAPK/ERK and PI3K/AKT by binding to the TrkB receptor, facilitating synaptic remodeling and plasticity in hippocampal neurons. Additionally, through a positive feedback mechanism, it further upregulates BDNF expression, improves mitochondrial function, and mediates the neuroplasticity regulation induced by exercise, alleviating depression [127,128]. An experiment on the elderly showed that exercise can activate the ERK downstream signaling pathway by increasing the expression of BDNF and its receptor TrkB in the hippocampus and prefrontal cortex, thereby inhibiting depressive-like behavior [129]. Research by Aguiar et al. found that 6 weeks of voluntary wheel running enhanced brain mitochondrial activity and upregulated the mRNA expression of BDNF, Glial cell line-derived neurotrophic factor (GDNF), TFAM, and Ndufa6 (mitochondrial complex I subunit), thereby improving neural plasticity and producing antidepressant effects [23]. Other studies have found that Post-Traumatic Stress Disorder (PTSD) is caused by hippocampal mitochondrial dysfunction, leading to issues such as anxiety and depression. Exercise can alleviate PTSD and improve mental disorders and cognitive dysfunction by increasing BDNF synthesis, improving mitochondrial function, and enhancing neural plasticity [130]. In summary, exercise activates the expression of the downstream gene BDNF through the CaMKII-CREB and MAPK signaling pathways, improves mitochondrial function, enhances neural plasticity, and alleviates depressive-like behaviors. Currently, there is limited research on how exercise alleviates depression by enhancing neural plasticity through improving mitochondrial function. Although it has been shown that exercise can improve mitochondrial function and enhance neural plasticity by upregulating BDNF expression, the temporal changes behind these mechanisms, the dynamic interactions between mitochondrial energy metabolism and neural plasticity during and after exercise, have not been fully clarified. For example, how do mitochondria quickly adjust energy supply during exercise to support immediate changes related to neural plasticity, such as enhanced synaptic transmission and altered neuronal excitability? Additionally, during the recovery period after exercise, how do these two factors work together to promote long-term adaptive changes in neural structure and function? Furthermore, the Wnt/β-catenin signaling pathway plays an important role in neural development and plasticity [131], but there is currently a lack of in-depth research on whether and how exercise can regulate the Wnt/β-catenin signaling pathway in neural cells, thereby promoting the proliferation and differentiation of neural stem cells, affecting neural plasticity and mitochondrial function, and improving depression (Figure 4).

## 5. Discussion

Exercise can serve as an effective complementary alternative to pharmacological or psychological treatments for depression. This study primarily focuses on how exercise alleviates depression by improving mitochondrial energy metabolism, but exercise can also improve depression through several other potential mechanisms. For instance, exercise can alleviate depression by influencing neurotransmitters, cytokines, and more. Swimming, in particular, enhances the sensitivity of serotonin 5-HT2 receptors and postsynaptic 5-HT1A receptors, increases levels of serotonin (5-HT) and 5-hydroxyindoleacetic acid (5-HIAA), and boosts the activity of tryptophan hydroxylase (TPH) and serotonin levels, thereby producing an antidepressant effect. Additionally, after exercise, the ratio of tryptophan to large neutral amino acids (TRP/LNAA) and the ratio of tryptophan to branched-chain amino acids (BCAA) increase, along with the upregulation of M5 receptors and nicotinic acetylcholine receptor α7 (nAChRα7) expression, significantly enhancing dopamine and acetylcholine levels, which in turn improves depression [132]. Another study found that the excessive activation of the HPA axis caused by abnormal secretion of glucocorticoids in patients with depression is considered an important pathogenic factor. Exercise alleviates depression by upregulating the expression of glucocorticoid receptors (GR) and increasing mRNA levels in brain regions such as the hippocampus, thereby restoring the negative feedback regulation function of the HPA axis [133,134]. In addition to the physiological mechanisms discussed above, exercise can also improve depression through psychological mechanisms such as enhancing self-efficacy, self-control, sense of belonging, and cognitive dissonance regulation [135,136].

The impact of mitochondrial energy metabolism on the improvement of depression through exercise is regulated by different types and durations of exercise. An experiment involving 61 college students randomly assigned to a high-intensity interval training group (HIT), a moderate-intensity continuous training group (MCT), and a control group (CON) over a period of six weeks found that MCT can alleviate neuroinflammation by reducing TNF-α levels, thereby improving mitochondrial energy metabolism and alleviating depression [23]. However, there are still contradictory results. Other studies have found that HIT can inhibit the production of TNF-α by inducing the release of IL-6, thereby producing an antidepressant effect that is superior to that generated by MCT [137]. Eight weeks of intense exercise effectively increased the levels of PGC-1α and mtDNA in the brains of mice [138], but it also led to an increase in the degree of apoptosis and oxidative damage [139]. Moderate aerobic exercise (60 min each time, 3 times a week, for 24 weeks) can promote mitochondrial biogenesis and produce antidepressant effects by increasing the activity of the IGF-I and PGC-1α pathways. Moderate-intensity swimming can enhance the expression of BDNF and its receptor TrkB in the hippocampus and prefrontal cortex, promote neurogenesis in the hippocampus, and improve mitochondrial function, thereby alleviating depressive-like behavior in mice [132]. Eight weeks of resistance exercise (RE) improves neuronal injury and synaptic plasticity in mice through different signaling pathways such as IGF-1, mTOR, and Akt, alleviating depressive-like behavior [140]. A recent study found that combined aerobic and resistance exercise (AERE) is more effective than resistance exercise (RE), yoga, and qigong in promoting the expression of BDNF and alleviating depression [141]. It is noteworthy that aerobic exercise can effectively promote neuroplasticity and BDNF expression, but its effects may depend more on the cumulative effect of exercise duration rather than the intensity of a single session [142]. Therefore, maintaining long-term moderate-intensity aerobic exercise is the best way to mediate improvements in mitochondrial energy metabolism to alleviate depression.

Current research on the mechanisms of mitochondrial energy metabolism regulation in depression shows certain heterogeneity in different stress models and brain region specificity. Studies have found that the knockout of SIRT1 in cortical and hippocampal glutamatergic neurons significantly reduces mitochondrial density and inhibits mitochondrial biogenesis, thereby inducing depressive-like behavior in male mice. However, the activation of SIRT1 can promote mitochondrial biogenesis and exhibit antidepressant effects [100]. However, other studies have found that chronic social defeat stress (CSDS) leads to an upregulation of SIRT1 expression in the nucleus accumbens, thereby mediating depressive-like behavior [143]. The differences present in different brain regions may be due to the variations in specific signaling pathways of SIRT1 in those regions. In the prefrontal cortex, SIRT1 may exert an antidepressant effect by increasing mitochondrial ATP production and the activity of glutamatergic neurons through the SIRT1-PGC-1α-BDNF signaling pathway [144]. Chronic stress induces the overexpression of SIRT1 in the nucleus accumbens, leading to enhanced transcriptional activity of FOXO3, which promotes increased apoptosis and reduced neuronal excitability, resulting in depressive-like behavior [145]. In addition, research shows that the expression levels of PGC-1α and NRF1 in the hippocampus of rats subjected to an acute restraint stress model are significantly higher than those in the control group [146]. Recent studies have shown that the expression of PGC-1α mRNA in the prefrontal cortex and hippocampal tissue is significantly downregulated in a depression model induced by prenatal stress [35]. The differences in research results caused by different stress models may be due to acute restraint stress, which, due to its short-term and intense stress characteristics, activates the body’s adaptive responses. This can enhance mitochondrial biogenesis and energy metabolism by activating signaling pathways such as AMPK and SIRT1, leading to the upregulation of PGC-1α and NRF1 expression, helping cells cope with stress responses [147]. In contrast, prenatal stress, characterized by its long-term and chronic nature, inhibits the expression of PGC-1α by activating glucocorticoid receptors (GR) and inflammatory signaling pathways (such as NF-κB), resulting in impaired mitochondrial function and increased oxidative stress [35]. It is worth noting that the heterogeneity of the regulation of mitochondrial energy metabolism-related signaling pathways caused by different exercise programs on depression-related outcomes has not been reported, and it is worth further exploration in the future.

CUMS is a relatively mature depression model compared to other stress models. It can induce long-term behavioral disturbances that are similar to the symptoms of clinical depression [92]. However, due to the multifactorial nature of depression, which involves the interaction of genetic, environmental, and psychological factors, the construction of animal models for depression is primarily based on stress exposure [2], making it difficult to fully simulate the pathological mechanisms of human depression. Human depression involves multiple brain regions (such as the hippocampus, prefrontal cortex, and amygdala) that have different functions. For example, the prefrontal cortex plays a key role in emotional regulation in humans, but it is not fully developed in rodents, which may lead to challenges in directly translating research findings related to relevant signaling pathways (such as SIRT1-PGC-1α) to clinical applications [148,149]. This limitation is also one of the constraints of this study.

Currently, research on how exercise improves mitochondrial energy metabolism to alleviate depression is still insufficient, and there are several issues that warrant further investigation: (1) Many factors can lead to abnormal mitochondrial energy metabolism that mediates the onset of depression, but this study did not delve into the specific mechanisms of differential expression of mitochondrial-related proteins in clinical and animal models, different types of depression, and various brain regions. (2) This research did not clarify the differences in the results of mitochondrial energy metabolism in exercise-induced improvement of depression under different types of exercise, intensity, individual differences, and duration, which is extremely important for the formulation of exercise prescriptions. (3) There is a need to further explore potential mechanisms and targets, focusing on new molecular targets such as mTOR, DRP1, NAD+, and P53, and their specific roles in exercise-induced improvements in mitochondrial energy metabolism, as well as elucidating the molecular mechanisms involved.

## 6. Conclusions

**Figure 5:**
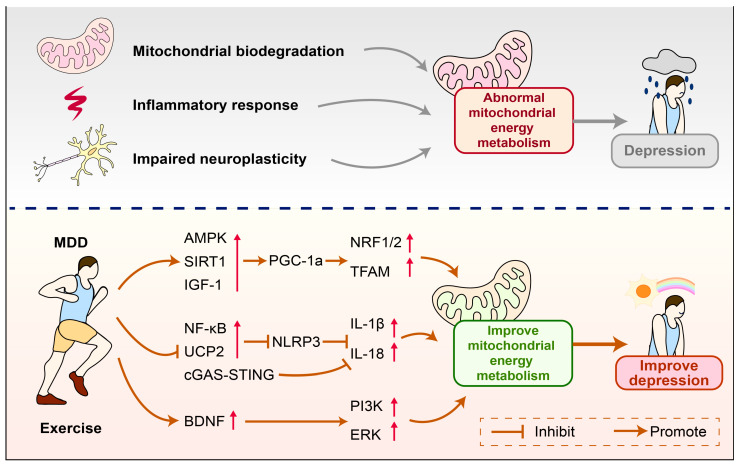
How exercise improves mitochondrial energy metabolism to alleviate depression. Reduced mitochondrial biogenesis, immune inflammation, and impaired neuroplasticity can lead to abnormal mitochondrial energy metabolism, which in turn mediates the occurrence of depression. Exercise can improve abnormal mitochondrial energy metabolism and alleviate depression by regulating the expression of related molecules. AMPK—AMP-activated protein kinase; PGC-1α—Peroxisome proliferator-activated receptor gamma coactivator 1-alpha; IGF-1—Insulin-like Growth Factor 1; SIRT1—Sirtuin 1; NRF1—Nuclear respiratory factor 1; NRF2—Nuclear factor erythroid 2-related factor 2; Transcription factor A, mitochondrial; NF-κB—Nuclear Factor kappa-light-chain-enhancer of activated B cells; UCP2—Uncoupling protein 2; cGAS—Cyclic GMP-AMP Synthase; STING—Stimulator of Interferon Genes; IL-18—Interleukin 18; IL-1β—Interleukin-1 beta; NLRP3—NOD-like receptor family pyrin domain containing; BDNF—Brain-derived neurotrophic factor; PI3K—Phosphatidylinositol 3-Kinase; ERK—Extracellular signal-regulated kinase.

Currently, the exact biological mechanisms of depression have not been fully elucidated. Existing evidence suggests that mitochondria play a key role in the onset and development of depression. This article reveals the potential mechanisms of mitochondrial energy metabolism in mediating the occurrence of depression and indicates that exercise is an important regulatory pathway. The molecular mechanisms are closely related to how exercise regulates the NLRP3 inflammasome to inhibit immune inflammatory responses, promotes the expression of PGC-1α to regulate mitochondrial biogenesis and improve mitochondrial energy metabolism, and upregulates the expression of BDNF to enhance mitochondrial function and thereby increase neural plasticity. This review provides new perspectives on the potential mechanisms regulating depression and offers references for how exercise can improve the onset and development of depression (Figure 5).
